# Two New *Novophytoptus* Species (Acariformes, Eriophyoidea) from Chile and a Novel Hypothesis on the Functioning of the Eriophyoid Digestive System †

**DOI:** 10.3390/ani16193083

**Published:** 2026-09-30

**Authors:** Philipp E. Chetverikov

**Affiliations:** Zoological Institute of Russian Academy of Sciences, Universitetskaya Nab., 1, St. Petersburg 199034, Russia; pchetverikov@zin.ru

**Keywords:** Apiaceae, Monocotyloidea, host shift, endoparasite, parenchymal necrosis, oesophagus, salivary glands, osmosis, transpiration, synergy

## Abstract

Endoparasitism is a highly specialized form of symbiosis, prominently manifested among phytophagous eriophyoid mites in the genus *Novophytoptus* Roivainen 1947. These mites have been traditionally thought to be restricted to the subepidermal lacunae of herbaceous monocots of the order Poales, which they penetrate to feed and reproduce. Based on surveys in Chile, two new species of *Novophytoptus* are described: *N. cyperi* **n. sp.** from *Cyperus eragrostis* (Cyperaceae) and *N. eryngii* **n. sp.** from *Eryngium paniculatum* (Apiaceae), with the latter representing the first record of *Novophytoptus* from a eudicot host. In accordance with the concept of “ecological fitting”, this unexpected association can be explained by the morphological similarity of *E. paniculatum* leaves with sedges, suggesting that convergent host morphology may drive host-switching between phylogenetically distant plant taxa across Eriophyoidea. Beyond taxonomy, modeling of *Novophytoptus* revealed a high volumetric disproportion (211-fold) between the microcapillary oesophagus and the massive midgut, along with a high body surface-area-to-volume ratio (~0.134 µm^−1^). Combined with structural constraints of musculature and intestine morphology, these findings challenge the traditional concept of mechanical pharyngeal pumping in Eriophyoidea. Alternatively, fluid ingestion is hypothesized to be driven by a synergy of an endodermal osmotic gradient and cuticular transpiration.

## Simple Summary

Eriophyoid mites are an economically important group of plant-feeding mites. Most known species feed on the epidermis, while a few have evolved as endoparasites that feed on subepidermal tissues. For decades, the endoparasitic genus *Novophytoptus* was considered exclusively associated with sedges, rushes, and grasses (all belonging to monocotyledons). Within these hosts, the mites live inside air cavities of leaves and stems. This paper reports two new species discovered in Chile, including the first record of this genus on a dicotyledonous host, *Eryngium paniculatum*, which mimics sedge morphology. Examinations of *Novophytoptus* mites using confocal laser scanning microscopy, coupled with a brief analysis of specialized literature on eriophyoid anatomy, revealed several structural and topographical constraints challenging the traditional mechanical pharyngeal pumping model of fluid ingestion in eriophyoid mites. A novel hypothesis assuming a synergistic interplay of osmotic forces in the intestine and cuticular transpiration is proposed to address these constraints.

## 1. Introduction

The superfamily Eriophyoidea is an ancient lineage of phytoparasitic acariform mites [1,2]. Their long history of colonizing higher vascular land plants dates back to the mid-Devonian, presumably stemming from an ancestral association with plant roots and mycorrhiza, followed by a transition to feeding on the tissues of above-ground vegetative and generative plant organs [3,4,5]. The unique Triassic amber fossils of eriophyoid-like plant-feeding mites (the triasacaroids sensu [2]) and the molecularly proven sister relationship between Eriophyoidea and soil-inhabiting mites of the family Nematalycidae make the Eriophyoidea an important member of time-calibrated arachnid phylogenies [5]. Testing evolutionary hypotheses within Eriophyoidea is impeded by a remarkably low ratio of described species relative to their estimated total diversity [6,7]. Furthermore, out of ~300 genera, only a few constitute truly monophyletic taxa, while most represent para- or polyphyletic “wastebaskets” [8,9,10] that are poorly, if ever, supported by reliable synapomorphies.

*Novophytoptus* Roivainen 1947 (Phytoptidae) is a rare, morphologically distinct, and molecularly supported monophyletic genus [11], providing a unique opportunity to investigate coevolutionary mite–host patterns. Although *Novophytoptus* has been found on every searched continent—with the exception of currently unsampled Australia—its global diversity remains poorly understood despite a clear dominance in European records that is mainly explained by biased sampling [12]. Intensive faunistic surveys are essential to capture the major lineages of intrageneric divergence prior to performing robust phylogenetic analyses of *Novophytoptus*.

Current evidence suggests that mites of this genus are restricted to the subepidermal leaf and stem lacunae of herbaceous Poales (primarily sedges, rushes, and grasses), which they penetrate to feed and reproduce [13,14]. Morphologically, *Novophytoptus* is remarkable for its very long, narrow, worm-like body, prognathous gnathosoma, and a highly reduced chaetome (lacking the gnathosomal setae *d*, leg setae *bv* I and *bv* II, tarsal solenidion *φ* I, and opisthosomal setae *c1*) in comparison to all other phytoptid genera. Additionally, mites of this genus possess extremely long prodorsal shield setae *sc* reaching up to ~100 µm in some species—a unique feature within Phytoptidae, with convergently long *sc* known only in a few genera of Eriophyidae (e.g., in some species of *Aceria*, *Aculus*, *Tetra*, or *Paraphytoptus*) and in some *Nalepella* (Nalepellidae). *Novophytoptus* also exhibits remarkably large, sac-shaped spermathecae and external genitalia that are notably shifted caudad, with ~15 coxigenital annuli situated between the basal genital coverflap and the coxae [6]. Although the functional role of these morphological transformations is still unclear, they likely evolved as adaptations to an endoparasitic lifestyle within subepidermal plant tissues [15]. Another interesting question concerns the anatomical modifications correlated with the body elongation in *Novophytoptus* that would, primarily, affect the structure of the alimentary tract, which must topographically stretch to span the entire distance from the anterior to the posterior extremity.

In this paper, I describe two new *Novophytoptus* species collected during 2025–2026 from Central Chile, including the first species of this genus discovered on a eudicot host. In addition to the taxonomy and a discussion of this unique host association, I report confocal laser scanning microscopy (CLSM) observations of *Novophytoptus*, revealing the topography of the internal structures involved in the feeding process in eriophyoid mites. By comparing these findings with anatomical data from the literature, I discuss how the classical mechanism explaining fluid transport through the alimentary canal may be impeded by anatomical constraints in Eriophyoidea, and propose a new hypothesis describing an alternative mechanism driving food ingestion into the midgut.

## 2. Materials and Methods

**Collection, morphological measurements and sequencing.** Plant samples were collected in February and March 2025 and 2026 in Curicó and near Vilches and Licantén in the Maule Region of Central Chile (Table 1). Leaf epidermis was removed with forceps, and eriophyoid mites were collected from subepidermal tissues using a fine minuten pin and a stereomicroscope. They were mounted in Hoyer’s medium and cleared on a heating block at 96 °C for 3 h [16,17]. Some material was stored in Eppendorf tubes in 96% ethanol in a freezer at −25 °C for subsequent DNA extraction, PCR, and sequencing of two marker genes (28S D1D2 rDNA and mitochondrial COI) using previously described protocols [11]. The slide-mounted specimens were examined with phase-contrast light microscopy (PC LM) and differential interference contrast light microscopy (DIC LM). In the species descriptions, measurements of the holotype (female) are presented along with ranges based on paratypes; for males, only ranges are given. All measurements are given in micrometers (μm) as lengths unless stated otherwise. Classification and terminology of eriophyoid external morphology and genital anatomy follow those of [1,6,18].

**Confocal microscopy.** Exoskeletons of *Novophytoptus* specimens mounted in Hoyer’s medium were investigated using confocal laser scanning microscopy (CLSM) with a Leica TCS SP2 spectral confocal and multiphoton system (Leica Microsystems GmbH, Wetzlar, Germany), following previously described microscope settings [18]. Image stacks were rendered three-dimensionally using Amira^®^ v.5.3.2 reconstruction software (FEI Visualization Science Group, Hillsboro, OR, USA). Maximum intensity projections (MIPs) and volume-rendered images (VRIs) were obtained to illustrate the topography of cuticular genital structures, the oesophagus, and the canals of prosomal glands.

**Surface area and volume estimations.** The mite’s vermiform body was approximated as a cylinder of length *L* and radius *R* to evaluate its volume *V* and surface area *S*. The additional volume and surface area contributed by the opisthosomal annuli were accounted for by modeling them as *N* circular folds of height *H* and width *W*.

The total surface area was calculated as *S* = 2π*RL* + 2π*R*^2^ + 2π*N*[(*R* + *H*)^2^ − *R*^2^)], where 2π*RL* + 2π*R*^2^ represents the total surface area of the smooth cylinder, and the final term accounts for the lateral walls of the *N* individual annuli (comprising 2*N* surfaces).

The total volume of the mite’s body was calculated by summing the volume of the base cylinder and the cumulative volume of the protruding annuli as follows: *V* = π*R*^2^*L* + π*NW*[(*R* + *H*)^2^ − *R*^2^)].

The surface-area-to-volume ratio (*SVR*) was calculated as *SVR* = *S*/*V*.

The oesophagus was modeled as a perfect hollow cylinder of the radius *r* and length *l*. Its internal volume was calculated as *V*(*oes*) = π*r*^2^*l*.

Following literature data [19,20,21,22], the midgut was assumed to occupy approximately 50% of the posterior half of the body volume; therefore, the midgut volume (*Vm*) was calculated as 0.25 × *V*, corresponding to 25% of the total body volume. The volume of the midgut lumen (*Vml*) was calculated using a conservative estimate from SEM micrographs published by previous authors, assuming its diameter accounts for ~40% of the entire midgut diameter, which yields a lumen volume equal to 16% of the midgut volume (*Vml* = 0.16 × *Vm*).

## 3. Results

### 3.1. Systematics

Superfamily Eriophyoidea Nalepa 1898

Family Phytoptidae Murray 1877

Subfamily Novophytoptinae Roivainen 1953

Genus *Novophytoptus* Roivainen 1947

#### 3.1.1. Description of *Novophytoptus cyperi* **n. sp.** (Figure 1 and Figure 2)

**FEMALE holotype (*n* = 11)**. Body vermiform, 278 (244–292), 46 (44–48) wide at the level of setae *c2*. **Prodorsal shield** subpentagonal, 24 (21–26), 24 (22–26) wide, with two ocellus-like spots in epicoxal area, and clusters of microtubercles anterior and lateral to the *ve* tubercles. Median line of prodorsal shield distinct, complete, and entire. Admedian lines complete, converging in the posterior third to form a single thick ridge. Three to five short, thin submedian lines present between the admedian line and the *ve* tubercle in the anterior half of the shield. Lateral areas between the *ve* and *sc* tubercles on each side with three to five faint, short lines. Distinct transverse line joining the admedian line medially and preceding the sc tubercle laterally on each side of shield. A subtriangular area, delimited by these lines, the tubercles of *sc*, and the first opisthosomal annulus, with sparse microtubercles. A small, arc-shaped line flanking a suboval area with the finest microtubercles located between the posterior ends of the admedian lines and the first dorsal opisthosomal annulus. Setae *ve* 11 (9–14), directed anterolaterad, tubercles 11 (10–11) apart; *sc* 57 (54–61), directed posterolaterad, tubercles 16 (14–18) apart. Distance between tubercles *ve*–*sc* 15 (15–17).

**Figure 1:**
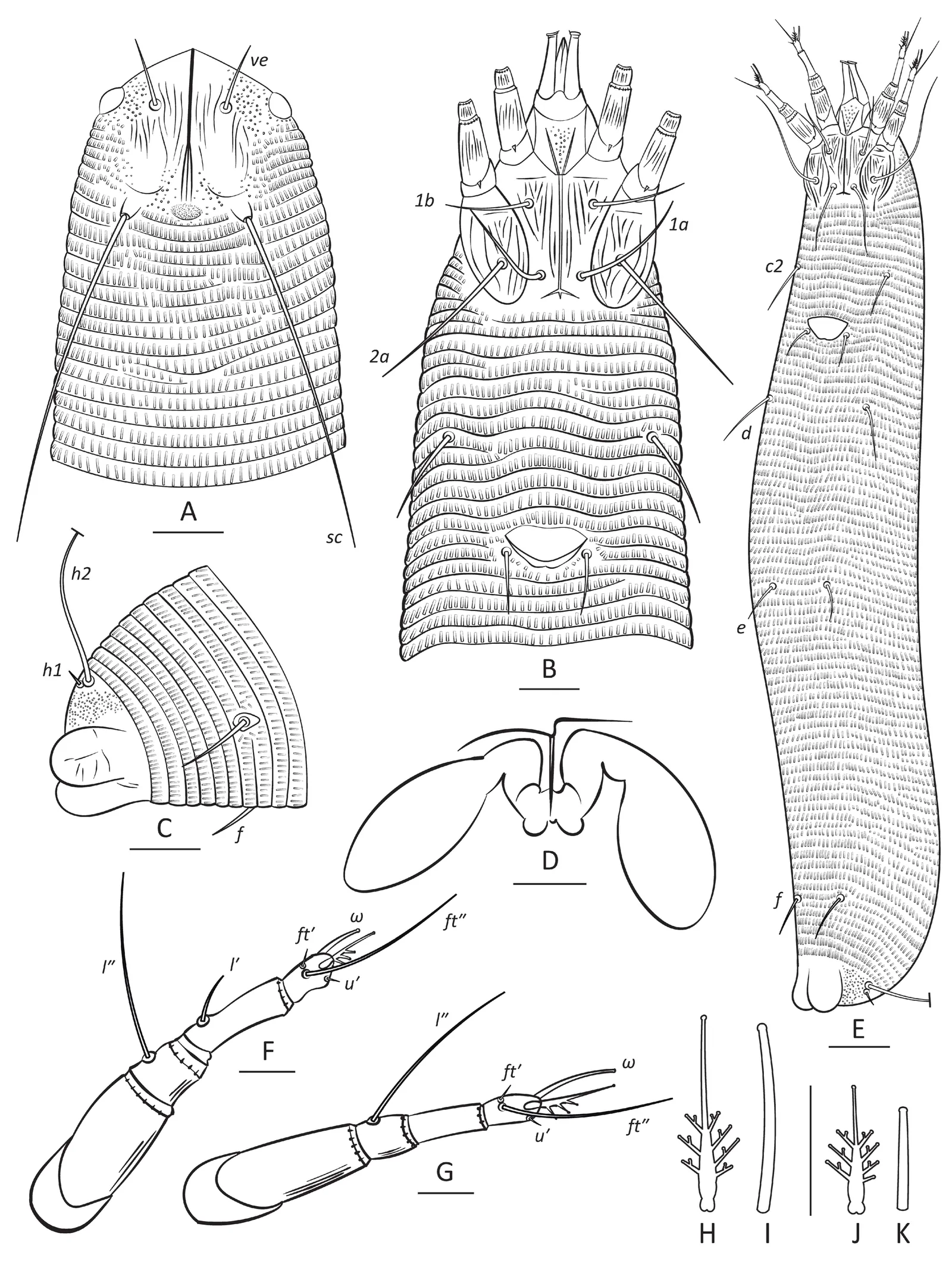
Drawings of *Novophytoptus cyperi* **n. sp.** (**A**)—prodorsal shield, (**B**)—coxigenital area, (**C**)—telosome, (**D**)—female internal genitalia, (**E**)—ventral view of entire mite, (**F**)—leg I, (**G**)—leg II, (**H**)—empodium II, (**I**)—tarsal solenidion II, (**J**)—empodium I, (**K**)—tarsal solenidion I. Scale bar: (**A**,**B**)—10 µm; (**C**)—15 µm; (**D**,**F**–**K**)—15 µm; (**E**)—20 µm.

**Figure 2:**
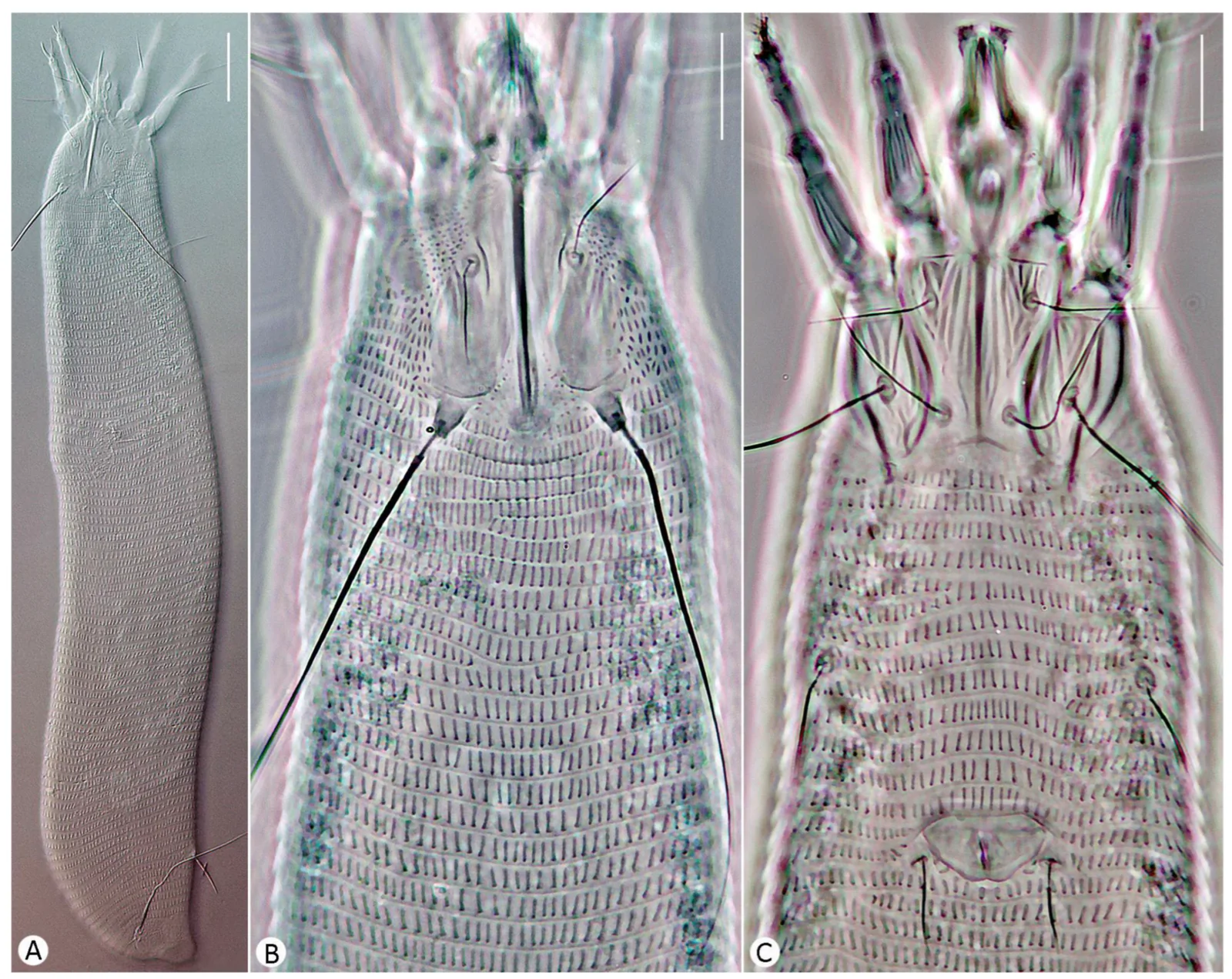
DIC LM (**A**) and PC LM (**B**,**C**) photographs of females of *Novophytoptus cyperi* **n. sp.** (**A**)—dorsal view of entire mite, (**B**)—dorsal view of prodorsal shield and anterior part of opisthosoma, (**C**)—ventral view of prosoma and coxigenital area. Scale bar: (**A**)—20 µm, (**B**,**C**)—10 µm.

**Gnathosoma** elongate, directed straight forward at slightly ventral angle, 23 (21–24); pedipalp coxal seta *ep* 2 (2–3), pedipalp genual seta *d* absent, subapical pedipalp tarsal seta *ν* 0.5 (0.5–1). Suboral plate with two diverging ridges forming a V-shaped figure and microtubercles between them.

**Leg I** 29 (28–30), tarsus 4 (3–5), *u′* 2 (1–2)*, ft′* 3 (2–3), *ft″* 10 (9–11), *ω* 4 (3–4) with tiny spherical knob, empodium I 4/4-rayed, 5 (4–6), each ray with one short secondary branch, the medial ray of the terminal pair 2–3 times longer than lateral ray; tibia 9 (8–9), ventrally striated, *l′* 4 (3–5), genu 5 (4–6), ventrally striated, *l″* 13 (11–14), femur 11 (10–12), ventrally striated, *bv* absent. **Leg II** 28 (27–29), tarsus 4 (3–4), *u′* 2 (1–3)*, ft′* 3 (2–3), *ft″* 15 (12–17), *ω* 7 (7–9) with tiny spherical knob, empodium II 4/4-rayed, 7 (6–8), each ray with one short secondary branch, the medial ray of the terminal pair 4–6 times longer than lateral ray; tibia 7 (6–7), smooth ventrally, genu 4 (3–4), ventrally striated, *l″* 14 (13–16), femur 10 (10–12), ventrally striated, *bv* absent.

**Coxal plates I and II** ornamented with longitudinal ridges. Prosternal apodeme inversely T-shaped, 19 (17–19). Setae *1b* 12 (9–14), 11 (10–11) apart; *1a* 22 (18–24), 7 (6–7) apart; *2a* 41 (35–44), 19 (18–21) apart; 13 (12–13) coxigenital annuli before epigynium. **Genital coverflap** semi-circular, smooth, 9 (8–10), 13 (12–15) wide; setae *3a* 9 (8–11), 13 (11–14) apart. **Internal genitalia (*n* = 6)**. Spermatheca sac-shaped, large, 11–13, 7–8 wide; spermathecal tube with narrower medial and swollen lateral segments, 5–6, longitudinal bridge 7–10, anterior genital apodeme 12–15 wide.

**Opisthosoma** vermiform, slightly expanded caudally, with 90 (82–98) dorsal and 78 (73–86) ventral annuli with elongated microtubercles. Lateral surface of caudal lobe with a group of small round microtubercles. Setal lengths: *c2* 13 (12–16), *d* 21 (19–24), *e* 9 (8–11), *f* 14 (13–15), *h1* 4 (3–5), *h2* 63 (59–69); 12 (11–13) annuli from rear shield margin to *c2*; 13 (11–15) annuli between *c2*–*d*; 19 (18–20) annuli between *d* and *e*; 34 (30–36) annuli between *e* and *f*; 7 (6–7) annuli between *f* and *h1*.

**GenBank data.** PZ798889 (*D1-D2 28S rRNA*, 1029 bp), PZ794033 (*COI*, 624 bp).

**Remarks**. Both accession numbers correspond to sequences obtained from the DNA isolate d1100, which originated from the type population of *N. cyperi***n. sp.** from the type locality in Curicó. For the additional population of *N. cyperi* **n. sp.** from Vilches (Table 2), amplification of the *COI* gene was unsuccessful, while the sequence of the *D1D2 28S* gene was 100% identical to that from the type population.

**Host plant**. *Cyperus eragrostis* Lam. (Cyperaceae).

**Figure 3:**
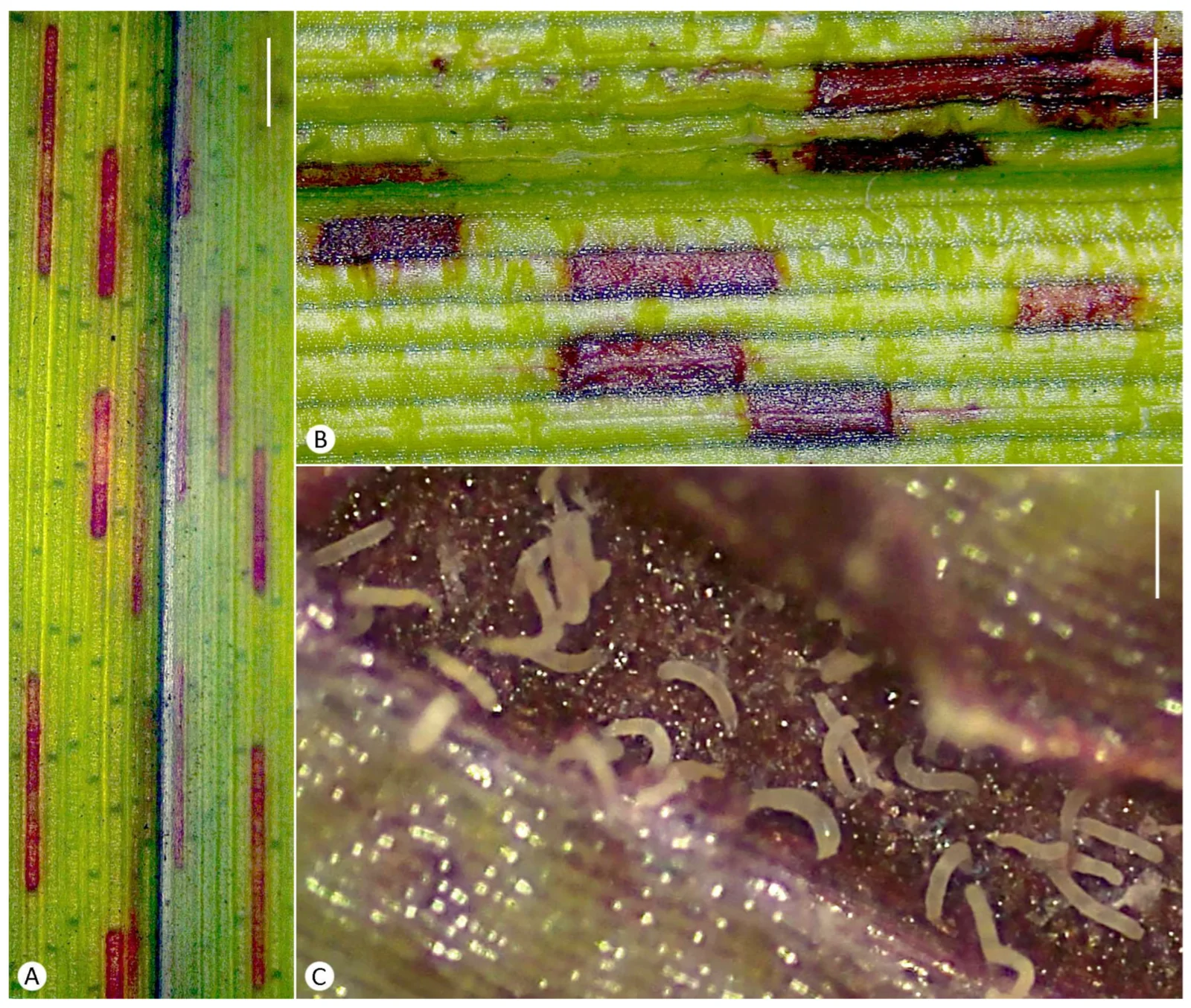
Damage caused by *Novophytoptus cyperi* **n. sp.** to the leaves of *Cyperus eragrostis*. (**A**,**B**)—“brown stripes” on the leaves (adaxial (**A**) and abaxial (**B**) surfaces); (**C**)—mites in the air cavity between leaf veins (epidermis removed). Scale bar: (**A**)—5 mm; (**B**)—3 mm, (**C**)—0.3 mm.

**Relation to the host.** Mites form large colonies consisting of hundreds of individuals in subepidermal air cavities of leaves (Figure 3). Mites feed on parenchymal cells and cause their necrosis. The areas of necrotized internal tissues are visible as “brown stripes” between leaf veins.

**Type locality.** Maule Region, Curicó, Parque cerro Carlos Condell, 34°58′33.1″ S 71°13′45.3″ W.

**Type material**. Female holotype on slide v2124-1, 10 paratype females in slide series v2124. All specimens from type locality, collected on 26 February 2026, coll. P.E.Chetverikov. Type material is deposited in the Acarological collection of ZIN RAS. Some paratypes have also been deposited in Laboratorio Regional Entomologia Region del Maule, Servicio Agricola y Ganadero (SAG) in Curicó (Chile).

**Additional material.** Numerous adults and immatures in slide series v2093, v2094, and v2108 collected in CHILE: Maule Region, near Vilches, 35°33′24.0″ S 71°15′15.5″ W, 8 March 2025, coll. P.E.Chetverikov.

**Etymology.** The specific epithet *cyperi* is a noun in the genitive case derived from the generic name of the host plant, *Cyperus*.

**Differential diagnosis.** Besides *N. silvai* Flechtmann 2004 from *Cyperus giganteus* Vahl. (Cyperaceae) from South America, the new species is the second member of *Novophytoptus* associated with the plant genus *Cyperus* L. (Cyperaceae). According to the original description of *N. silvai*, mites of this species have a series of distinctive longitudinal lines on prodorsal shield between tubercles of *sc* and *ve* [13]; however, reinvestigation of the paratypes failed to detect these lines and revealed almost smooth prodorsal shield, possibly because of fading in mounting medium [12]. Main morphometric differences between *N. silvai* and *N. cyperi* **n. sp.** are in the number of ventral opisthosomal annuli and length of setae *sc* (Table 2). Among all known species of *Novophytoptus*, the new species is closest to *N. limpopoensis* Chetverikov et al. 2023 from a sedge *Carex spicatopaniculata* Boeckeler ex C.B.Clarke (Cyperaceae) from South Africa. The main differences are in the presence/absence of subtriangular microtuberculated area between tubercles of *sc*, length of setae *sc*, and shape of empodia I (Table 2).

#### 3.1.2. Description of *Novophytoptus eryngii* **n. sp.** (Figure 4 and Figure 5)

**FEMALE holotype (*n* = 10)**. Body vermiform, 295 (264–343), 39 (36–43) wide at the level of setae *c2*. **Prodorsal shield** subpentagonal, 30 (28–32), 31 (27–31) wide, with two ocellus-like spots lateral to setae *ve* and numerous microtubercles covering entire shield except the central area bearing longitudinal lines. Median line of prodorsal shield distinct, slightly sinuous or fragmented in some paratypes, present only in posterior half of the shield. Admedian lines complete, converging near the anterior margin of median line. Two short, straight submedian lines (I and II) present between the admedian line and the *ve* tubercle in the anterior half of the shield. Two short, oblique lines each followed but 2 to 3 short longitudinal ridges on each side of admedians at the level of the anterior margin of median line.

**Figure 4:**
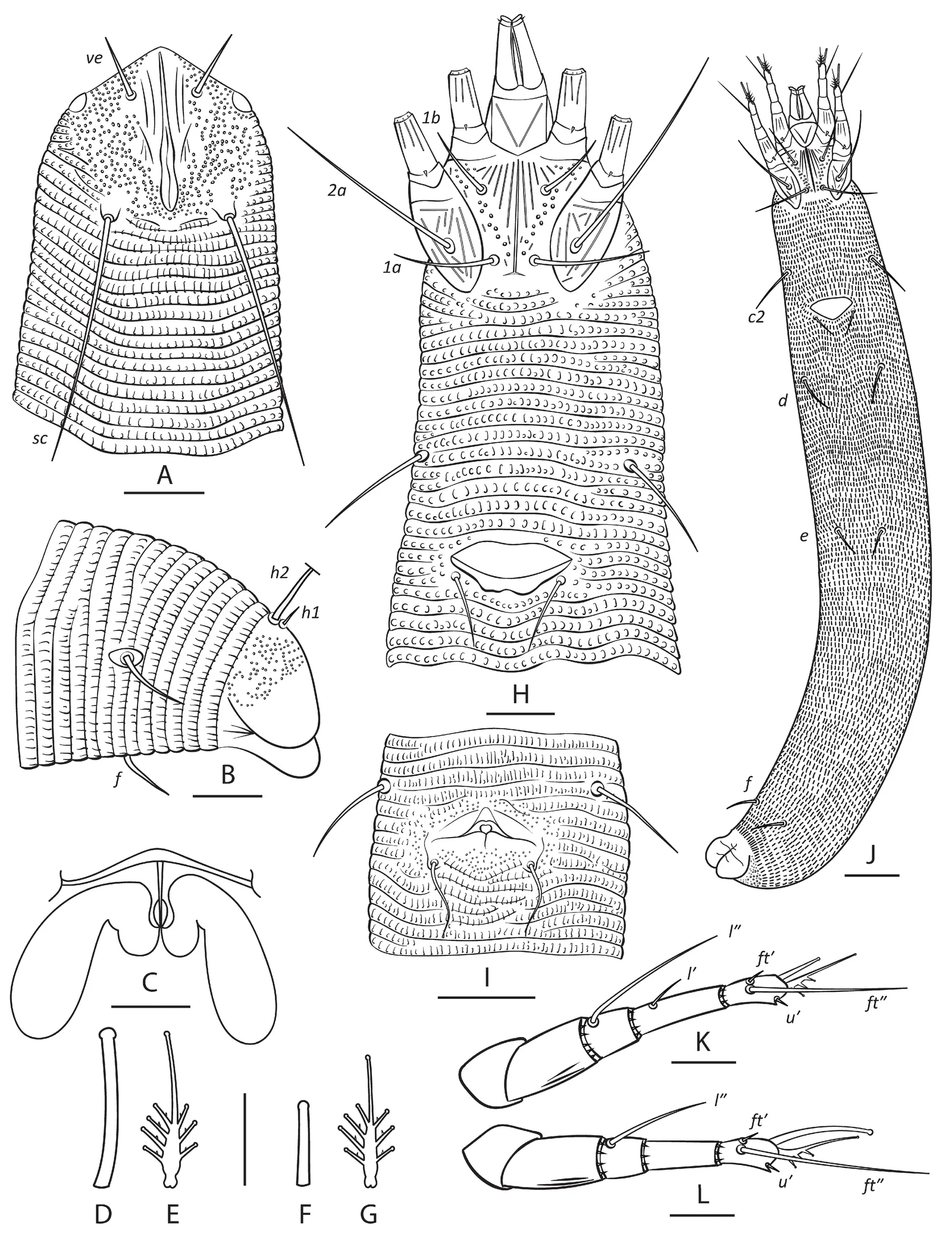
Drawings of *Novophytoptus eryngii* **n. sp.** (**A**)—prodorsal shield, (**B**)—telosome, (**C**)—female internal genitalia, (**D**)—tarsal solenidion II, (**E**)—empodium II, (**F**)—tarsal solenidion I, (**G**)—empodium I, (**H**)—coxigenital area, (**I**)—male genital area, (**J**)—ventral view of entire mite, (**K**)—leg I, (**L**)—leg II. Scale bar: (**A**,**B**,**H**)—10 µm; (**C**–**G**,**K**,**L**)—5 µm; (**I**)—15 µm. (**J**)—20 μm.

**Figure 5:**
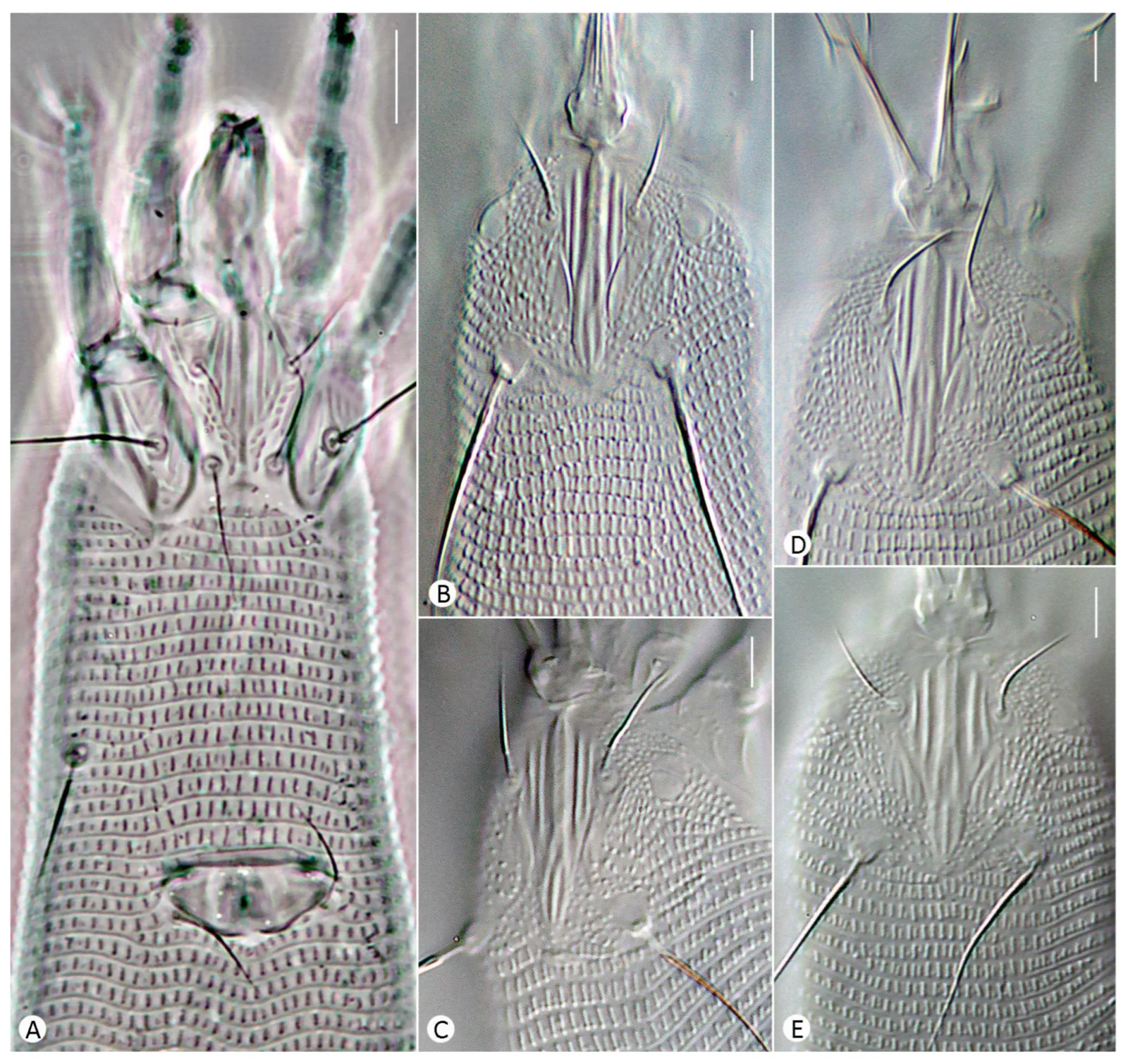
PC LM (**A**) and DIC LM (**B**–**E**) photographs of *Novophytoptus eryngii* **n. sp.** (**A**)—female coxigenital area; (**B**–**E**)—prodorsal shields in three females (**B**–**D**) and male (**E**). Scale bar: (**A**)—10 µm, (**B**–**E**)—5 µm.

Prodorsal shield subpentagonal, 30 (28–32), 31 (27–31) wide, with two ocellus-like spots lateral to setae *ve* and numerous microtubercles covering the entire shield except the central area bearing longitudinal lines. Median line of prodorsal shield distinct, slightly sinuous or fragmented in some paratypes, present only in the posterior half of the shield. Admedian lines complete, converging near the anterior margin of the median line. Two short, straight submedian lines (I and II) present between the admedian line and the *ve* tubercle in the anterior half of the shield. Two short, oblique lines each followed by 2 to 3 short longitudinal ridges on each side of the admedian lines at the level of the anterior margin of the median line. Setae *ve* 12 (11–14), directed anterolaterad, tubercles 8 (8–9) apart; *sc* 37 (33–42) long, directed posterolaterad, tubercles 16 (15–19) apart. Distance between tubercles *ve*–*sc* 15 (15–16).

**Gnathosoma** elongate, directed straight forward at slightly ventral angle, 19 (18–20); pedipalp coxal seta *ep* 2 (1–2), pedipalp genual seta *d* absent, subapical pedipalp tarsal seta *ν* 1 (0.5–1). Suboral plate smooth, with two faint diverging ridges forming a V-shaped figure.

**Leg I** 27 (25–28), tarsus 5 (5–6), *u′* 3 (2–3)*, ft′* 2 (1–3), *ft″* 13 (11–15), *ω* 4 (4–6) with tiny spherical knob, empodium I 4/4-rayed, 5 (5–6), each ray with one short secondary branch, the medial ray of the terminal pair 2–3 times longer than lateral ray; tibia 6 (6–7), ventrally smooth or with very faint 1 to 3 longitudinal ridges, *l′* 3 (2–4), genu 5 (4–5), *l″* 13 (11–15), femur 8 (7–8), with 3 longitudinal striae ventrally, *bv* absent. **Leg II** 26 (24–27), tarsus 5 (4–5), *u′* 3 (2–3)*, ft′* 3 (2–3), *ft″* 16 (14–18), *ω* 8 (7–9) with tiny spherical knob, empodium II 4/4-rayed, 7 (6–8), each ray with one short secondary branch, the medial ray of the terminal pair 4–5 times longer than lateral ray; tibia 5 (4–6), genu 4 (4–5), *l″* 13 (12–15), femur 7 (7–8), with 3 longitudinal striae ventrally, *bv* absent.

**Coxal plates I** ornamented with symmetrically distributed subparallel ridges anteriorly and large subspherical microtubercles distributed along the lateral coxal margin. Coxal plates II ornamented with irregular ridges and devoid of microtubercles. Prosternal apodeme inversely T-shaped, 16 (15–17). Setae *1b* 10 (8–11), 9 (9–10) apart; *1a* 19 (17–20), 6 (5–6) apart; *2a* 31 (25–35), 17 (16–18) apart; 16 (15–17) coxigenital annuli before epigynium. **Genital coverflap** narrow, rounded posteriorly, smooth, 9 (9–10), 16 (14–17) wide; setae *3a* 12 (11–14), 14 (13–14) apart. **Internal genitalia (*n* = 7)**. Spermatheca elongate, suboval, 10–14, 4–6 wide; spermathecal tube with smaller medial and larger swollen lateral segments, 4–6, longitudinal bridge short, 5–7, anterior genital apodeme 13–15 wide.

**Opisthosoma** vermiform, slightly expanded caudally, with 125 (121–129) dorsal and 116 (111–121) ventral annuli with elongated microtubercles. Lateral surface of caudal lobe with a group of small round microtubercles. Setal lengths: *c2* 17 (15–19), *d* 14 (13–16), *e* 11 (10–12), *f* 10 (9–11), *h1* 7 (7–8), *h2* 54 (40–63); 12 (11–13) annuli from rear shield margin to *c2*; 20 (18–21) annuli between *c2*–*d*; 27 (25–29) annuli between *d* and *e*; 51 (48–54) annuli between *e* and *f*; 7 (6–7) annuli between *f* and *h1*.

**MALE (*n* = 5)**. Body vermiform, 254–318, 36–41 wide at the level of setae *c2*. **Prodorsal shield** subpentagonal, 20–23, 20–23 wide, ornamented similarly to females. Setae *ve* 8–11, directed anterolaterad, tubercles 9–11 apart; *sc* 20–24, directed posterolaterad, tubercles 15–17 apart. Distance between tubercles *ve*–*sc* 14–15. **Gnathosoma** elongate, directed straight forward at slightly ventral angle, 18–20; pedipalp coxal seta *ep* 1–2, pedipalp genual seta *d* absent, subapical pedipalp tarsal seta *ν* 0.5–1. Suboral plate subtriangular, smooth, with two ridges forming V-shaped figure.

**Leg I** 22–25, tarsus 4–5, *u′* 1–2*, ft′* 2–3, *ft″* 10–14, *ω* 3–4 with tiny spherical knob, empodium I 4/4-rayed, 3–4, each ray with one short secondary branch, terminal paired rays short and equally long; tibia 5–6, *l′* 2–3, genu 3–4, *l″* 11–13, femur 7–8, *bv* absent. **Leg II** 21–24, tarsus 4–5, *u′* 1–3*, ft′* 2–3, *ft″* 12–14, *ω* 7–9 with tiny spherical knob, empodium II 4/4-rayed, 3–4, similarly shaped to empodium I; tibia 4–5, genu 3–4, *l″* 10–12, femur 7–8, *bv* absent. Coxal plates ornamented similarly to those of females. Prosternal apodeme inversely T-shaped, 11–13. Setae *1b* 9–11, 8–10 apart; *1a* 14–17, 5–7 apart; *2a* 24–29, 14–18 apart; 13–15 coxigenital annuli before epiandrium.

**Genital area** 9–12, 14–16 wide, with a small, papilla-shaped genital cone in the center, preceded by a narrow transverse genital slit and a thin cuticular subtriangular flap; setae *3a* 14–16, 16–18 apart, setae *eu* non-apparent.

**Opisthosoma** with 105–123 dorsal and 101–116 ventral annuli with elongated microtubercles. Lateral surface of caudal lobe with a group of small, round microtubercles. Setal lengths: *c2* 12–16, *d* 10–12, *e* 6–10, *f* 8–10, *h1* 6–7, *h2* 29–35; 12–14 annuli from rear shield margin to *c2*; 16–18 annuli between *c2*–*d*; 23–28 annuli between *d* and *e*; 43–50 annuli between *e* and *f*; 5–6 annuli between *f* and *h1*.

**GenBank data.** PZ798888 (*D1-D2 28S rRNA*, 1023 bp); PZ794095 and PZ794096 (*COI*, 1198 bp).

**Remarks.** The sequences of the *D1-D2 28S* gene obtained from DNA isolates d1111 (from the type population in Curicó) and d1105 (from the additional population from Licantén) of *N. eryngii* **n. sp.** were identical (Table 1). Amplification of the COI gene produced sequences of equal length (PZ794095 for isolate d1105 and PZ794096 for isolate d1111) differing at 10 nucleotide positions (p-distance = 0.008, K2P = 0.008). All of these differences represented synonymous nucleotide substitutions in the third codon position.

**Host plant**. *Eryngium paniculatum* Delaroche (eudicots: Apiales, Apiaceae).

**Figure 6:**
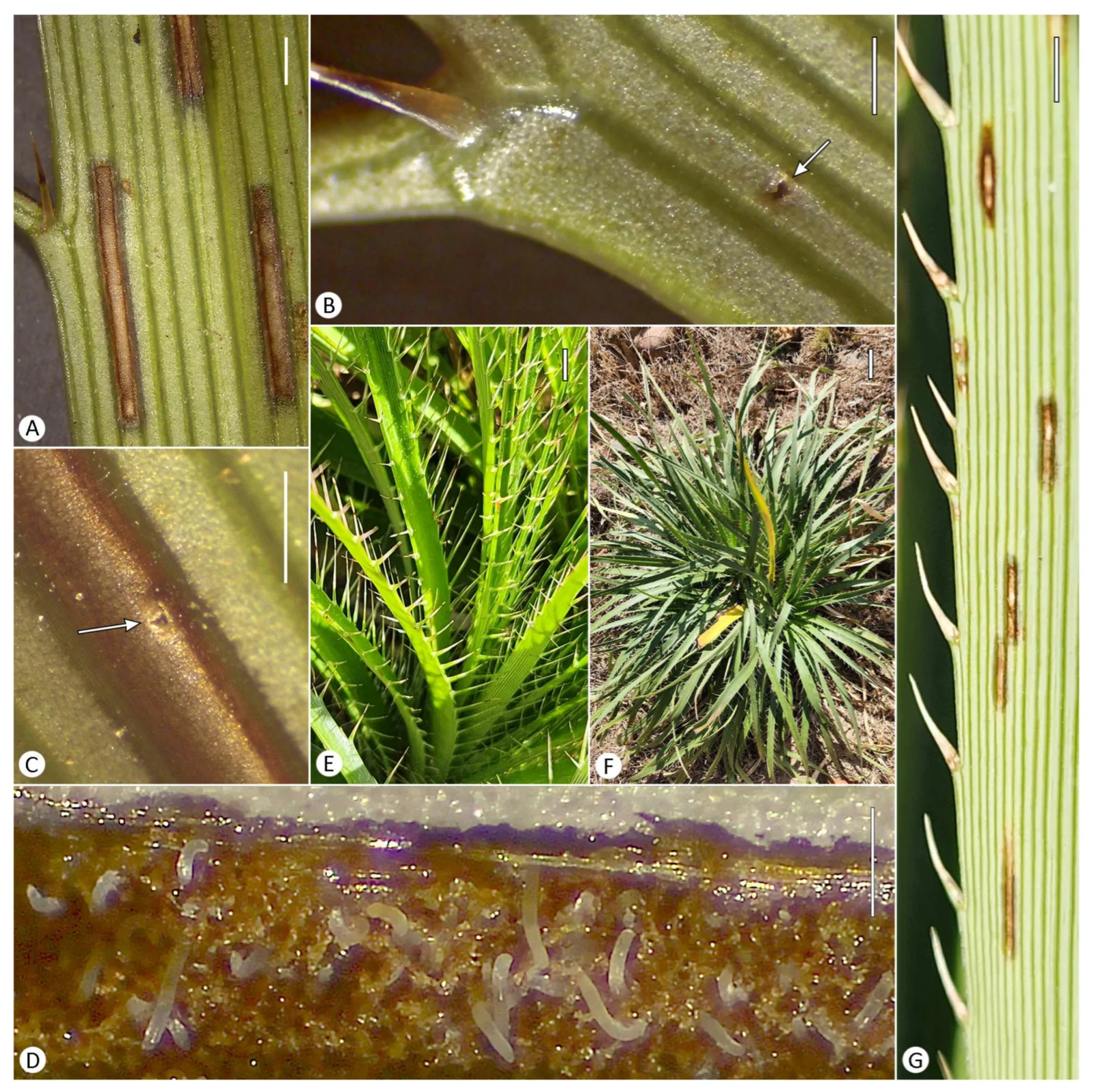
Damage caused by the mite *Novophytoptus eryngii* **n. sp.** to the leaves of *Eryngium paniculatum*. (**A**,**G**)—“brown stripes” on leaves marking the areas where mites feed under the epidermis ((**A**)—adaxial, (**G**)—abaxial); (**B**,**C**)—presumed entrance (**B**) and exit (**C**) openings (arrows); (**D**)—mites in air cavities between necrotized mesophyll cells inside the leaf (adaxial epidermis removed); (**E**,**F**)—two plants of *E. paniculatum* sampled in different localities in Central Chile ((**E**)—in Curicó, (**F**)—near Licantén). *Remarks*: The two yellow leaves in the central part of the plant shown in Figure 6F were especially heavily infested with mites. Scale bar: (**A**)—2 mm; (**B**,**C**)—1 mm, (**D**)—0.3 mm, (**E**)—2 cm, (**F**)—10 cm, (**G**)—5 mm.

**Relation to the host.** Up to now *N. eryngii* **n. sp.** is the only *Novophytoptus* species known from eudicot hosts. Similar to monocot-associated species of *Novophytoptus*, *N. eryngii* **n. sp.** are subepidermal endoparasites. They penetrate internal leaf tissues through circular holes in the epidermis, live in the intercellular spaces and feed on mesophyll cells causing local necrosis, which is visible as “brown stripes” between leaf veins (Figure 6).

**Type locality.** CHILE: Maule Region, Curico, near exit of Parque cerro Carlos Condell, 34°58′41.9″ S 71°13′53.9″ W.

**Type material**. Female holotype on slide v2145-18, 16 paratype females and 7 males in slide series v2145. All specimens from type locality, collected on 4 March 2026, coll. P.E.Chetverikov. Type material is deposited in the Acarological collection of ZIN RAS. Some paratypes have also been deposited in Laboratorio Regional Entomologia Region del Maule, Servicio Agricola y Ganadero (SAG) in Curicó (Chile).

**Additional material.** Numerous adults and immatures in slide series v2134 collected in CHILE: Maule Region, about 6 km north to Licantén, along the road J-810, 34°56′44.8″ S 71°58′30.6″ W, 1 March 2026, coll. P.E.Chetverikov.

**Etymology.** The specific epithet *eryngii* is a noun in the genitive case derived from the generic name of the host plant, *Eryngium*.

**Differential diagnosis.** The new species differs from all currently described *Novophytoptus* spp. by having coxae I ornamented with subspherical microtubercles arranged in two symmetrical, diverging rows (whereas such microtubercles are absent on the coxae of other species), and by the densely microtuberculated lateral fields of the prodorsal shield that are devoid of lines (lateral fields are non-microtuberculated and bear more or less distinct short lines in all species except *N. longissimus* Chetverikov and Petanović 2016). Among *Novophytoptus* species, the new species is closest to *N. longissimus*. The main differences are in body size, the ornamentation of the prodorsal shield and coxae, and the lengths of the leg and opisthosomal setae (Table 3).

### 3.2. CLSM Observations

**Figure 7:**
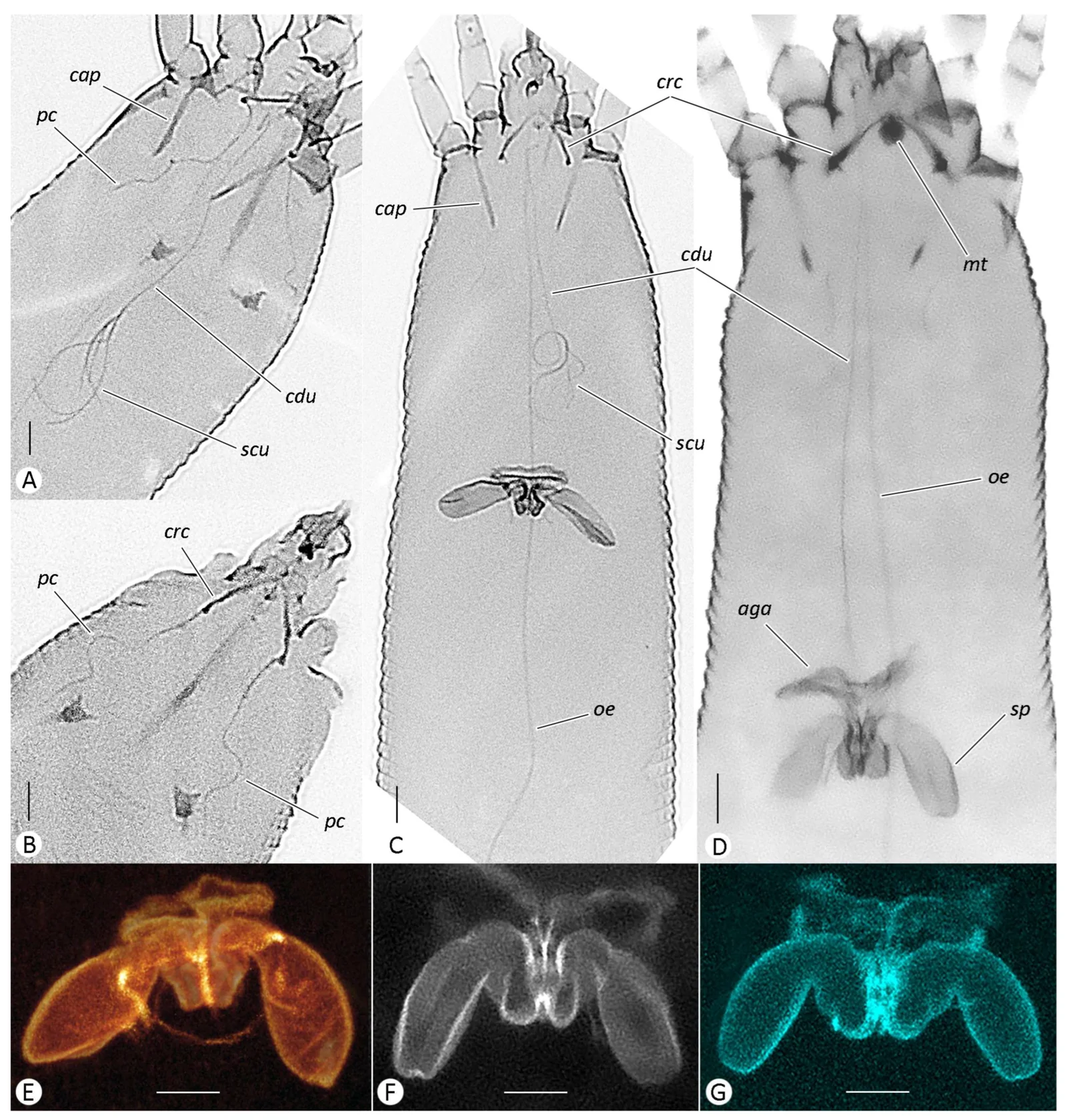
CLSM images (**A**–**D**)—black and white inverted MIP; (**E**–**G**)—volume rendering) showing relative position of cuticular elements of prosomal glands and oesophagus (**A**–**D**) and female internal genitalia (**E**,**F**) in *Novophytoptus cyperi* **n. sp.** (**A**–**C**,**E**) and *N. eryngii* **n. sp.** (**D**,**F**,**G**). Notations: *aga*—anterior genital apodeme, *cap*—coxal apodeme, *cdu*—common duct of unpaired gland, *crc*—cervical canal, mt—motivator, *oe*—oesophagus, *pc*—podocephalic canal, *scu*—secretory canaliculi of unpaired gland, *sp*—spermatheca. Scale bar = 5 µm.

Under a violet laser (405 nm), the following autofluorescing internal cuticular structures were observed in all investigated females of *N. cyperi* **n. sp.** (*n* = 6) and *N. eryngii* **n. sp.** (*n* = 7): the female internal genitalia, including the paired spermathecae, spermathecal tubes, longitudinal bridge, and anterior genital apodeme; the coxal and palpal apodemes; a subspherical motivator; a capillary-like oesophagus; and the canals of the prosomal glands (Figure 7). In some incompletely cleared females, the outlines of partially dissolved soft-tissue internal organs, including the ovary, central nervous system, and voluminous midgut occupying roughly 50% of the posterior half of the body volume, were also detected. The relative position of all listed structures largely corresponds to that reported by previous authors [21,23], yet some new observational data were obtained.

Posterior to the gnathosoma, at the level of coxae I, two distinct, straight cuticular canals—herein named the *cervical canals*—converge anteriorly to open beneath the motivator in the subcheliceral plate (the cervix). Their posterior funnel-shaped expansions anchor the thin, sinuous podocephalic canals. These canals run beneath, and are probably attached to, the dorsal shield; they then diverge, making two sharp bends anterior to the tubercles of *sc* setae, before terminating in the lateral cuticle where only traces of the dissolved soft tissue of the podocephalic glands rarely remain visible.

In all examined specimens, the cuticular elements of an unpaired gland were visualized in the medial region of the anterior body. These elements comprise secretory canaliculi that are intertwined into a cluster, frequently forming loops and rings, which coalesce into a linear cuticular common duct. This duct runs medially forward and passes through the motivator. On dorsoventral projections, the common duct often overlaps with the oesophagus because they run one above the other and share a similar, constant diameter of ~1 µm. Within this pair of cuticular tubes, the oesophagus initially occupies a ventral position; however, near the anterior genital apodeme, it ascends dorsally to run closer to the dorsal cuticle. It terminates in the posterior half of the middle third of the body, where a small terminal expansion—resembling the terminal funnels of the cervical canals—is visible, likely corresponding to the oesophageal valve [21] at the transition into the midgut. The total length of the oesophagus ranged from 140 to 170 µm.

Averaged morphometric values obtained during CLSM investigation for *Novophytoptus* mites (body length L = 300 µm, radius R = 25 µm; N = 100; oesophagus radius *r* = 0.5 µm, length *l* = 150 µm) yield a total body surface area (S) of 83,095.1 µm^2^ and a volume (V) of 621,092.9 µm^3^. The surface-area-to-volume ratio (SVR) is ~0.134 µm^−1^, while the oesophagus volume (*Voes*) is 117.8 µm^3^, the midgut volume (*Vm*) is 155,273.2 µm^3^, and the midgut lumen volume (*Vml*) is 24,843.7 µm^3^.

## 4. Discussion

**Ecological fitting and host colonization in *Novophytoptus*.** In this paper, two new species of *Novophytoptus* (*N. cyperi* **n. sp.** and *N. eryngii* **n. sp.**) are described from Chile—a biogeographically unique region, poorly explored by eriophyidologists, that retains an ancient Gondwanan floral and faunal connection. Among them, *N. cyperi* **n. sp.** is a typical representative of the genus associated with herbaceous monocots, possessing a classical relation to its hosts via feeding and reproducing inside leaf air cavities [13]. Also being a subepidermal endoparasite, *N. eryngii* **n. sp.** is unique in that it is the first member of the genus found associated with Apiaceae, one of the highly derived lineages within the core eudicots [24]. Although monophyletic generalist species and genera are known among Eriophyoidea [25,26], none except *Leipothrix* Keifer 1966 (Eriophyidae) have been shown to exploit both eudicots and monocots. With the discovery of *N. eryngii* **n. sp.**, *Novophytoptus* becomes the only confirmed monophyletic genus within Phytoptidae recorded from both of these major angiosperm clades. Given its deeply specialized endoparasitic lifestyle, implying a tight adaptation to host plant anatomy and tissue characteristics, this broad host range is highly unexpected.

Remarkably, *E. paniculatum*, the apiacean host of *N. eryngii* **n. sp.** (as well as most other members of the *Eryngium* subgenus *Monocotyloidea*), demonstrates high morphological convergence with monocots [27,28]. Its leaves externally and anatomically resemble those of agaves, yuccas, or large sedges; they are long and sword-shaped, with parallel venation. Similar to Cyperaceae and Juncaceae (the putative primary hosts of *Novophytoptus*), the mesophyll between the longitudinal veins in Monocotyloidea contains air cavities divided into chambers by thin transverse plates of parenchymal cells—the spaces where *N. eryngii* **n. sp.** was found feeding and reproducing. The anatomical similarity to sedges most likely facilitated the host shift of *Novophytoptus* to *Eryngium*, overriding the substantial phylogenetic distance between these plant taxa. This hypothesis aligns with the evolutionary scenario of “ecological fitting” [29], specifically its form of “resource tracking” [30], and implies that the association of *Novophytoptus* with Apiaceae is secondary. Confirmation of this hypothesis by future molecular phylogenies would provide a solid basis for a more generalized evolutionary framework, suggesting that host morphological convergence drives host-switching between phylogenetically distant plant taxa across the entire Eriophyoidea. Additionally, the discovered association with *Eryngium* indicates that future surveys for new *Novophytoptus* species should also target eudicot taxa, primarily those exhibiting a convergent resemblance to monocots. This includes not only certain species of Apiaceae with linear or septate leaves, such as *Bupleurum*, *Eryngium*, *Lilaeopsis*, and *Oenanthe* [31], but also representatives from other families (e.g., Asteraceae, Campanulaceae), which share a similar sedge-like leaf structure with subepidermal air cavities.

**A new hypothesis on the functioning of the eriophyoid digestive system.** Nuzzaci and Alberti [21] provided a generalized scheme of the eriophyoid digestive tract, concluding that the transport of ingested material is achieved by the pharyngeal pumping apparatus. It comprises a sickle-shaped pharynx with a heavily sclerotized ventral and flexible dorsal cuticle, and dilator muscles that elevate the roof of the pharynx to create a sucking force supported by the movements of the peripheral body muscles. This concept assumes a high pharyngeal pumping capacity to push discrete portions of plant sap through the oesophagus via external muscular peristalsis, much like the active piston-like cycles observed in fluid-feeding insects [32]. However, this classical mechanism is impeded by several anatomical constraints in eriophyoid mites. First, their cuticular oesophagus forms a remarkably long and narrow microcapillary (~150 × 1 μm in *Novophytoptus*), which according to Poiseuille’s law creates immense hydraulic resistance [33,34]. Second, eriophyoids lack a developed visceral muscular coat around the oesophagus except for a putative sphincter in the area where the oesophagus protrudes via a valve into the midgut lumen [21], rendering peristaltic waves along the rigid chitinous lining functionally unfeasible. Third, the small pharynx lacks a robust muscular or valvular compression framework to actively push fluid; its muscles function exclusively as dilators to create suction, while the subsequent expulsion relies entirely on the passive, low-power elastic recoil of a thin, flexible dorsal cuticle [21]. Furthermore, their skeletal and peripheral musculatures are topographically non-specialized for managing localized gastrointestinal transit. Finally, despite ingesting large cumulative volumes of liquid sap over their lifespan, eriophyoid mites lack Malpighian tubules [21] and pronounced hindgut peristalsis, preventing voluminous defecation and in some taxa the anal opening is probably non-functioning [35].

These constraints might be resolved through a hypothetical mechanism rooted in the striking volume disparity between the oesophagus and midgut, as well as high surface-area-to-volume ratios, which likely contribute to osmosis and transpiration in Eriophyoidea. Indeed, modeling an average adult *Novophytoptus* specimen reveals that the tiny internal volume of the oesophagus abruptly transitions into the midgut exceeding the oesophagus by 211-fold in lumen volume. Given this topography and the abundance of cytological markers indicating intensive cellular transport within the midgut epithelium [21], the midgut may function as a powerful osmotic pump. Under this mechanism, fluid from the capillary-like oesophagus could be passively drawn to the midgut by a high osmotic potential constantly maintained by the anterior and posterior parts of the midgut operating in a coordinated manner, where an osmotic gradient generated in the anterior midgut drives reabsorption in the posterior midgut. Notably, the absence of a basal lamina between the epithelia of these regions, which are in physical contact with each other [21] (p. 132), could facilitate the internal circulation of osmo-active particles.

Complementing this internal drive, the body shape of eriophyoids provides a high surface-area-to-volume ratio (SVR). Based on the *Novophytoptus* model, approximating the body as a cylinder with annular folds yields a baseline SVR of ~0.134 µm^−1^, predetermining intensive transcuticular transpiration as postulated by previous authors [36]. Continuous passive evaporation of moisture through the thin integument removes water from the body, thereby stabilizing the internal osmotic suction. Importantly, this transcuticular transpiration may function as a dynamic autoregulatory mechanism driven by the unique longitudinal muscles of Eriophyoidea. Since the osmolarity of vacuolar sap fluctuates significantly even within a single host plant [37,38,39,40], the peripheral body muscles can reflexively adjust the distance between the opisthosomal annuli, altering the exposure of the thin interannular cuticle. This allows the mite to physically modulate its evaporative surface area [41], and, probably, adjust its internal osmotic potential to maintain a steady fluid current from the pierced plant cell directly into the oesophagus. Within the framework of this hypothesis, the role of the eriophyoid pharyngeal pump shifts from regulating discrete “gulps” and forcing fluid portions through the oesophagus to initiating the suction of a priming volume and modulating the initial fluid pressure as it begins to surge from the pierced plant cell into the alimentary canal. Finally, the coordinated contractions of the peripheral and dorsoventral muscles increase intra-prosomal hydrostatic pressure, likely driving the discharge of prosomal gland secretions.

## 5. Conclusions

Endoparasitism represents an evolutionary pathway for eriophyoid mites on higher vascular plants, as exemplified by the genus *Novophytoptus*, which exhibits a highly specialized host range and ecology, being restricted to subepidermal tissues of monocots. In addition to *N. cyperi* **n. sp.** from the widely distributed monocot *Cyperus eragrostis*, this study reports the first discovery of *Novophytoptus* (*N. eryngii* **n. sp.**) on a dicotyledonous host, *E. paniculatum*. Such a novel host association demonstrates that the host spectrum of highly specialized endoparasitic lineages of Eriophyoidea is not necessarily narrower or more restricted than that of their non-endoparasitic counterparts, thereby contrasting with the expectation that endoparasitism, as a highly specialized evolutionary pathway, entails closer intimacy and obligate dependence on specific hosts. In the case of *Eryngium*, the association with *Novophytoptus* was likely facilitated by the morphological convergence of *E. paniculatum* with monocots, which served as a crucial prerequisite for “ecological fitting” during *Novophytoptus* evolution.

Modeling of *Novophytoptus*, combined with a critical review of the literature, reveals anatomical constraints that undermine the traditional model of mechanical pharyngeal pumping in Eriophyoidea. To address these limitations, a novel hypothesis of a synergistic interplay between the midgut osmotic gradient and cuticular transpiration is proposed as an alternative mechanism for fluid ingestion. Testing this hypothesis in the future requires, first, fluid-dynamics biophysical modeling to calculate the mechanical forces and pressure drops across the microcapillary oesophagus under the traditional pharyngeal pumping model. This necessitates quantifying critical variables: total feeding time, cell sap viscosity, ingestion volume, pharynx capacity, and the muscle distension required for elastic cuticular recoil. Second, the osmotic pump hypothesis can be evaluated using artificial diets with varying osmolarities; if the midgut operates via osmotic suction, increased dietary osmolarity should inhibit fluid uptake by reducing the osmotic gradient. Third, the role of transcuticular transpiration as a hydrodynamic driver can be verified by monitoring feeding under controlled humidity, as a saturated (100% RH) atmosphere should decelerate the passive fluid current. Finally, high-resolution in vivo confocal or video microscopy can visualize pharyngeal and intestinal dynamics, clarifying whether muscle contractions correlate with the periodic expulsion of prosomal gland secretions.

## Figures and Tables

**Table 1:** Collecting data and accession numbers of partial COI and D1D2 28S sequences of two *Novophytoptus* species from Chile.

Mite Species	Host Plant	DNA Isolate	Collection Data	*COI*	*D1-D2 28S*
*N. cyperi* **n. sp.**	*Cyperus eragrostis* Lam. (Cyperaceae)	d878	CHILE: Maule Region, near Vilches, 35°33′24.0″ S 71°15′15.5″ W, 8 March 2025, coll. P.E.Chetverikov	no data	100% identity with PZ798889
		d1100 (type population)	CHILE: Maule Region, Curicó, Parque cerro Carlos Condell, 34°58′33.1″ S 71°13′45.3″ W, 26 February 2026, coll. P.E.Chetverikov	PZ794033 (624 bp)	PZ798889 (1029 bp)
*N. eryngii* **n. sp.**	*Eryngium paniculatum* Delaroche (Apiaceae)	d1105	CHILE: Maule Region, about 6 km north to Licantén, along the road J-810, 34°56′44.8″ S 71°58′30.6″ W, 1 March 2026, coll. P.E.Chetverikov	PZ794095 (1198 bp)	100% identity with PZ798888
		d1111 (type population)	CHILE: Maule Region, Curicó, near exit of Parque cerro Carlos Condell, 34°58′41.9″ S 71°13′53.9″ W, 4 March 2026, coll. coll. P.E.Chetverikov	PZ794096 (1198 bp)	PZ798888 (1023 bp)

**Table 2:** Morphological differences between three *Novophytoptus* species.

Character	Mite Species		
	*N. cyperi* n. sp.	*N. limpopoensis* Chetverikov et al. 2023	*N. silvai* Flechtmann 2004
Length of *sc*	54–61	65–83	40–44
Number of ventral annuli	73–86	72–85	63–73
Shape of empodium I	Asymmetrical (rays of the terminal pair are unequally long)	Symmetrical (rays of the terminal pair are subequally long)	Asymmetrical (rays of the terminal pair are unequally long)
Microtuberclated area between tubercles of *sc*	Present	Absent	Two groups of microtubercles mesal to the tubercles of sc, present in the drawings of the original description, but non-apparent in the paratypes [12].
Reference	This paper	[12]	[13]

**Table 3:** Morphological differences between *Novophytoptus eryngii* **n. sp.** and *N. longissimus* Chetverikov and Petanović 2016.

Character	Mite Species	
	*N. eryngii* n. sp.	*N. longissimus* Chetverikov and Petanović 2016
Number of longitudinal lines between tubercles of ve	6	About 15
Paired short oblique lines in the central area of prodorsal shield	Present	Absent
Median line of prodorsal shield	Present only in posterior half of prodorsal shield	As long as prodorsal shield
Microtubercles along lateral margin of coxae I	Present	Absent
Number of dorsal opisthosomal annuli	121–129	108–134
Number of ventral opisthosomal annuli	111–121	104–137
Length of *sc*	54–61	62–78
Length of *ve*	11–14	20–25
Shape of empodium I	Asymmetrical (rays of the terminal pair are unequally long)	Symmetrical (rays of the terminal pair are subequally long)
Reference	This paper	[14]

## Data Availability

All new DNA sequences obtained in this study have been deposited in the National Center for Biotechnology Information (NCBI) GenBank database (https://www.ncbi.nlm.nih.gov/genbank) (accessed on 26 August 2026).
